# Rethinking Chronic Wound Treatment: Unlocking the Potential of Combination Products for an Unmet Multifactorial Need: A Review Study

**DOI:** 10.1002/hsr2.71798

**Published:** 2026-02-08

**Authors:** Alberto Nicolás Ramos, Nicolás Cerusico, Romina Chavez‐Jara

**Affiliations:** ^1^ Institute of Pharmaceutical and Food Biotechnology (INBIOFAL) CONICET–National University of Tucuman San Miguel de Tucuman Tucuman Argentina

**Keywords:** chronic wounds, combination products, diabetic foot ulcers, pressure ulcers, venous ulcers, wound healing

## Abstract

**Background and Aims:**

Chronic wounds, including diabetic foot, venous, and pressure ulcers, remain a major unmet medical challenge due to their prevalence, severity, and the limited efficacy of current treatments. These wounds are inherently multifactorial, requiring simultaneous intervention across all chronicity‐inducing factors. Neither medical devices nor single pharmacological agents are sufficient, as they cannot comprehensively address the multiple therapeutic needs. This review aims to propose an integrative therapeutic approach capable of targeting all relevant mechanisms.

**Methods:**

A narrative review of the literature was conducted, analyzing over 100 peer‐reviewed articles on chronic wound pathophysiology and therapeutic strategies. Sources were identified through searches in PubMed, Scopus, and Web of Science, complemented by manual reference screening. Studies discussing the mechanisms of wound chronicity, as well as drugs and biologics with potential therapeutic activity, were included.

**Results:**

The analysis revealed that current therapeutic options, including devices, drugs, and biologics, address only isolated aspects of chronic wound pathophysiology. No single agent or device is capable of comprehensively targeting all relevant mechanisms. However, evidence suggests that combining already‐approved drugs and/or biologics may provide a synergistic effect, simultaneously targeting inflammation, infection, impaired angiogenesis, oxidative stress, and defective tissue remodeling. Importantly, the use of approved components leverages established pharmacological and safety profiles, potentially streamlining development and regulatory approval.

**Conclusion:**

A topical combination product integrating multiple agents offers a promising strategy to overcome the limitations of current treatments. Advances in the understanding of wound pathophysiology and the availability of diverse active molecules create new opportunities to design effective and holistic therapies. Such combination products could transform the management of chronic wounds and represent the next generation of treatment approaches.

## 1 Introduction

Chronic wounds, affecting approximately 1%–2% of the population in developed countries [1], a number expected to rise with the increasing prevalence of diabetes, obesity, and an aging population [1, 2, 3]. Their widespread occurrence, substantial impact on quality of life, and the limited effectiveness of current treatments [3] establish them as a significant unmet medical need [1, 2, 3, 4]. This highlights an urgent clinical and economic demand for more effective therapies, driving regulatory agencies, such as the FDA to prioritize innovative solutions for chronic, non‐healing wounds [1, 5].

Traditionally, chronic wounds are defined as wounds that fail to heal within a timeframe that would normally be sufficient for recovery [6]. There is no established consensus on the specific duration that constitutes chronicity, and these wounds are frequently managed as comorbid conditions [6]. Clinically, chronic wounds are characterized by their inability to progress through the normal stages of healing [7].

**FIGURE 1:**
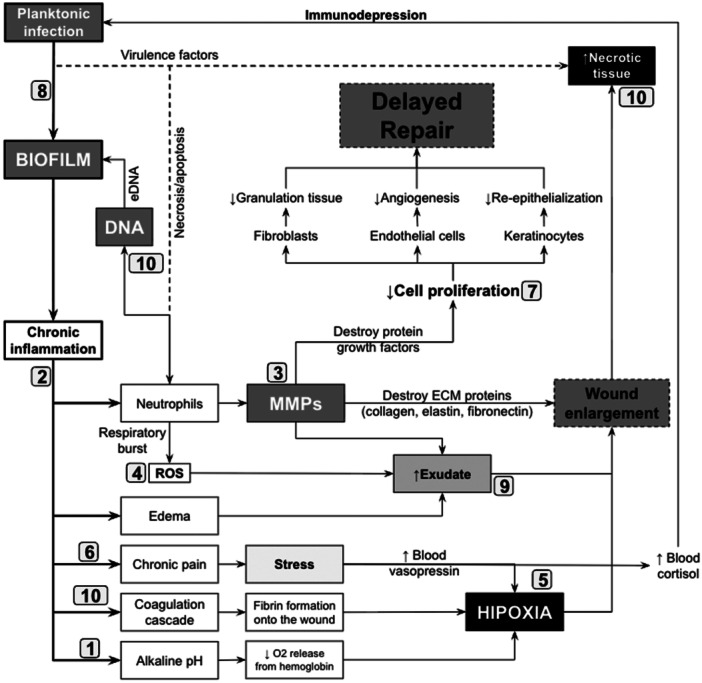
After ulceration, all chronic wounds exhibit common factors that drive the development of a unified pathophysiological model of chronification. Multiple interconnected deleterious feedback loops fuel the 10 chronicity‐inducing factors, resulting in two concurrent states: wound enlargement and delayed repair. Chronic infection (8) initiates chronic inflammation (2) and its associated effects, including neutrophil infiltration, edema, chronic pain (6), coagulation abnormalities (10), and an alkaline pH (1). Hypoxia (5) develops because of elevated vasopressin levels, impaired angiogenesis, pericapillary fibrin deposition, and reduced oxygen release from hemoglobin. Neutrophils attempting to phagocytose biofilm bacteria undergo apoptosis and/or necrosis, releasing DNA—subsequently utilized in the biofilm matrix—along with matrix metalloproteinases (MMPs) (3), reactive oxygen species (ROS) (4), and other harmful factors that accumulate in the exudate (9). MMPs degrade extracellular matrix (ECM) proteins, including collagen, elastin, and fibronectin, exacerbating wound enlargement. Combined with hypoxia and bacterial virulence factors, this degradation contributes to the accumulation of necrotic tissue (10). Additionally, matrix metalloproteinases (MMPs) degrade critical wound‐healing proteins, impairing cell proliferation (7), which hinders granulation tissue formation, angiogenesis, and re‐epithelialization—thereby perpetuating the non‐healing state.

The Wound Healing Society (WHS) classifies chronic wounds into three main categories: diabetic, pressure, and vascular ulcers [8]. The underlying pathology (diabetes, prostration, and vascular insufficiency) predisposes the skin and surrounding tissues to ulceration [9, 10, 11, 12]. Following ulceration, all chronic wounds share common chronicity‐inducing factors (CIFs) that allow for the development of a unified pathophysiological model of chronification (Figure 1) [13]. Collectively, CIFs sustain a vicious cycle of tissue destruction and impaired repair, resulting in the chronic non‐healing state. The CIFs are: (1) wound alkalinization [14], (2) chronic inflammation [15], (3) matrix metalloproteinases (MMPs) hyperactivity [16], (4) oxidative stress [13, 17], (5) hypoxia [18], (6) chronic pain [19], (7) impaired cellular proliferation and extracellular matrix (ECM) formation [20], (8) chronic infection [21], (9) excessive exudation [22], and (10) necrotic tissue accumulation [23].

Wound care experts recommend addressing all CIFs, leading to a consensus on the therapeutic needs required to improve outcomes (Table 1) [24, 25, 26, 27, 28]. All therapeutic needs should be applied simultaneously, as all CIFs are interconnected through multiple feedback loops (Figure 1). Targeting only one or a subset of these factors is insufficient, since the remaining pathways can restore the chronic state.

**TABLE 1:** Chronicity‐inducing factors, their respective therapeutic needs, and therapeutic agents recommended to achieve them.

Chronicity‐inducing factors	Therapeutic needs	Therapeutic mechanism	Therapeutic agent
1. Wound alkalinization	Acidification of the wound bed	Direct acidification to pH between 6.8 and 7.2	Organic acid
	Avoid wound alkalinization	Bacterial urease inhibition by cofactor chelation	Chelating agent
2. Chronic inflammation	Regulation of several pro‐inflammatory processes.	Pleiotropic mechanisms caused by acidification to pH 6.8–7.2	Organic acid + ascorbic acid
3. MMPs hyperactivity	Avoid ECM and growth factors destruction produced by MMPs	MMPs inhibition by acidification and cofactors chelation	Organic acid + chelating agent
4. Oxidative stress	Avoid tissue destruction produced by respiratory burst	Radical scavenging and antioxidation avoiding Fenton reaction	Free radical scavenger + chelating agent
5. Hypoxia	↑ O_2_ releasing from hemoglobin	Anti‐Bohr effect	Organic acid
	Vasodilation	eNOS stimulation	Lactic acid
	Angiogenesis	Stimulation of VEGF production	Lactic acid
	Elimination of aerobic bioburden	↓ Oxygen consumption	See point 8
6. Chronic pain	↓ Pain‐related stress	↓ Cortisol and vasopressin effects	Analgesic agent
7. Lack of cellular growth	ECM components formation	Synthesis of collagen and elastin	Lactic acid and ascorbic acid
		Elastase inhibition (acidification)	Organic acid
	↑ Proliferation of fibroblasts and keratinocytes	Acidification to pH 6.8–7.2	Organic acid
8. Infection elimination	Wide spectrum bacteriostatic and bactericide for planktonic bacteria	Combination of acid stress, lithic action, alteration of membrane and LPS structure, chelation of essential cations, and wound oxygenation	Organic acid + surfactant + chelating agent
	Wide spectrum biofilm inhibition	↓ Planktonic bacteria adhesion and proliferation	Organic acid
	Wide spectrum biofilm disruption	↓ EPS electrostatic interactions, cation chelation, ↓ superficial tension and eDNA destruction.	Organic acid + surfactant + chelating agent + DNase
9. Exudate management	Exudate absorption	Suppressing the action of deleterious components	Cellulose‐derived agent
10. Wound debridement	Activation of autolytic debriding	Acidification to pH 6.8–7.2	Organic acid
	Expose healthy, well‐perfused tissue that is able to proliferate and populate the wound bed	↓ Fibrin accumulation onto the wound	Chelating agent
		DNA gel destruction onto the wound	DNase

However, most available products are medical devices that address only a few therapeutic needs [29]. Combining medical devices also fails, as they cannot be applied simultaneously [30]. Even advanced therapies fail to ensure healing because they do not target all necessary factors [31, 32, 33, 34]. Treatment approaches vary by institution and clinician [31] although the most effective protocols typically combine patches, negative pressure, hyperbaric therapy, enzymatic debridement, tissue engineering, and growth factors, creating a significant economic burden for health systems [1, 6, 35]. Even so, nearly 60% of chronic wounds take more than a year to heal, with healing times ranging from 3 to 7 years in developing countries [1, 35] and often requiring amputation [35, 36].

As an effective treatment demands a comprehensive, multi‐targeted approach [37], it is necessary to use a combination of components. However, some challenges have restricted the adoption of combination products in this field. For example, multi‐component therapies must navigate multiple regulatory frameworks, and the lack of a standardized approach for coordinating multi‐factor interventions has slowed innovation.

We propose that a combination product integrating FDA‐approved drugs and biologics represents a viable strategy for chronic wound management. By leveraging existing approvals, development can be accelerated while maintaining safety and efficacy. Our research introduces a conceptual framework supporting the use of a single topical formulation capable of simultaneously targeting all CIFs in wound healing. This approach has the potential to transform current practice by providing a scientifically grounded, multi‐factorial intervention that lowers regulatory and financial barriers, ultimately improving patient outcomes and advancing the field of regenerative medicine.

## 2 Methods

This study is a narrative review conducted through a structured literature search. PubMed, Scopus, and Web of Science were searched for studies published between January 1990 and June 2025, using combinations of the following keywords: “chronic wounds,” “diabetic foot ulcer,” “pressure ulcer,” “venous ulcer,” “wound healing,” “oxidative stress,” “inflammation,” “infection,” “biofilm,” “angiogenesis,” “pH,” “hypoxia”, “debridement” and “treatment.” Additional references were identified through manual searches of the bibliographies of relevant articles.

Inclusion criteria were: (i) peer‐reviewed publications; (ii) studies addressing chronic wounds (including diabetic foot ulcers, venous ulcers, and pressure ulcers) or pathophysiological mechanisms relevant to wound chronicity; and (iii) original research articles, clinical studies, and review papers. Exclusion criteria included: (i) case reports and (ii) conference abstracts.

To guide the design of a potential combination product, we also reviewed the portfolio of FDA‐approved drugs and biologics with mechanisms of action relevant to chronic wound pathophysiology. These were identified through the FDA's Drugs@FDA database, regulatory reports, and published literature. Molecules and biologics were evaluated based on their safety profiles, regulatory status, and therapeutic potential to target CIFs. This evaluation may not capture all potential therapeutic candidates currently under investigation or approved by other regulatory agencies.

Extracted information was synthesized qualitatively and organized thematically according to CIFs, in order to map recurrent mechanisms of wound chronicity and identify therapeutic opportunities with FDA‐approved agents.

Physicochemical properties and formulation compatibility of the candidate molecules were assessed using data retrieved from PubChem, a public chemical database maintained by the National Center for Biotechnology Information (NCBI).

### 2.1 The Hypothesis

We hypothesize that it is feasible to design a comprehensive and practical solution for chronic wound management [38, 39] based on the following guiding principles:

a. All CIFs, and thus all therapeutic needs (Table 1), must be targeted concurrently.
b. The therapeutic solution should consist of a combination of drugs and/or biologics.
c. Each component should ideally exert multiple therapeutic effects and/or act synergistically with others, thereby minimizing the total number of agents required.
d. To reduce regulatory barriers, the combination should prioritize FDA‐ or other regulatory agency–approved drugs and biologics with well‐established safety, pharmacokinetic, pharmacodynamic, and immunogenicity profiles.
e. Therapeutic properties should be delivered locally and simultaneously through a semisolid topical formulation, which allows efficient incorporation of multiple agents and effective wound‐site delivery.

### 2.2 Evaluation of the Hypothesis: Choosing the Correct Molecules and/or Biologics

In the following section, we explore the factors contributing to wound chronification, emphasizing the therapeutic needs for effective treatment. By drawing on existing literature, we identify the most suitable components to address these needs while aligning with the principles of an effective solution as proposed in our hypothesis (Table 1).

### 2.3 Addressing Wound Alkalinization

**FIGURE 2:**
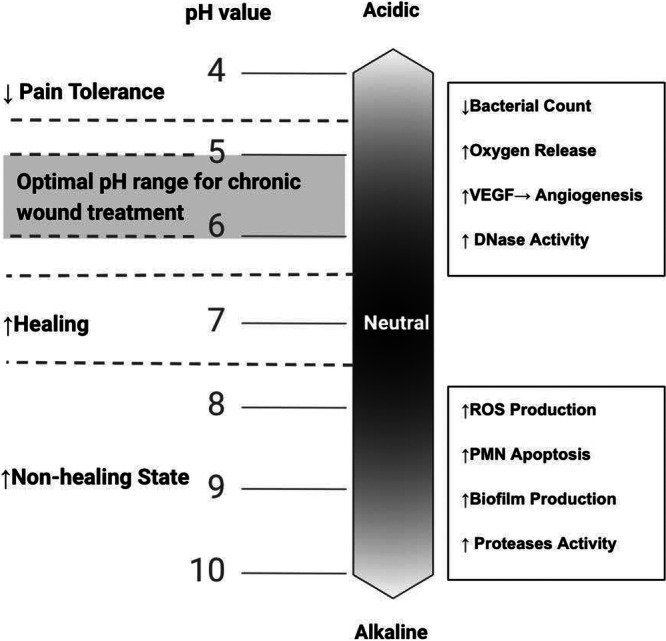
Wound healing process at different pH values. The optimal pH range for chronic wound treatment is highlighted as 5–6, where pain tolerance is maximized, angiogenesis and DNase activity are enhanced, and bacterial count is significantly reduced. In more alkaline conditions, the wound environment fosters increased production of reactive oxygen species (ROS), polymorphonuclear leukocyte (PMN) apoptosis, biofilm formation, and elevated protease activity, all of which contribute to a non‐healing state.

Wound healing is influenced by pH changes at every stage [9, 14, 40, 41, 42, 43]. Chronic wounds typically have a pH between 7.5 and 8.9, which slows healing compared to wounds closer to neutral pH [9, 42]. The optimal pH for healing ranges from 6.8 to 7.15 [44, 45], while injured skin tolerates pH 4.5–6.5, though pain increases significantly below pH 5.0 [45, 46]. Therefore, a product with a pH of 5.0–6.0 is ideal for promoting healing, as it can transition the alkaline wound environment to its optimal healing pH without causing significant pain (Figure 2). Moreover, acidification addresses multiple therapeutic needs, including resolving chronic inflammation, regulating MMP activity, enhancing oxygenation, stimulating cell proliferation, and controlling infection [9, 13, 14, 40, 41]. Reducing wound pH with organic carboxylic acids is a promising, low‐cost strategy due to their biocompatibility and low toxicity [14, 47]. Among options like lactic, acetic, citric, and malic acids, lactic acid stands out for its additional beneficial properties, as discussed in subsequent sections.

Additionally, some chelating agents like EDTA may prevent wound alkalinization by inhibiting urease, a bacterial nickel‐dependent metalloenzyme that hydrolyzes urea to ammonia, further disrupting the wound environment [48, 49].

### 2.4 Addressing Chronic Inflammation

Acidification of the wound bed enhances healing by modulating the immune response and transitioning the wound out of the chronic inflammatory stat [9, 42, 43, 49]. Chronic wounds are characterized by persistent inflammation and high leukocyte concentrations, which can be regulated by acidic pH levels. At a pH of 6.5–6.7, macrophage and PMN chemotaxis, along with leukocyte mobility, decrease, while lymphocyte motility improves, allowing the immune response to progress to a post‐inflammatory phase [41, 43]. Elevated apoptosis of activated Polymorphonuclears (PMNs) at the typical pH of chronic wounds (7.7–8.2) results in the release of harmful granules (MMPs, free radicals, etc.), contributing to tissue damage and chronic inflammation [50]. However, at a pH of 6.7–7.2, PMN apoptosis drops significantly [50]. Additionally, at pH levels of 5–6.7, the respiratory burst of PMNs following phagocytosis is reduced [41], and at pH 5.5, the production of α Tumoral Necrotic Factor (TNF‐α) [51, 52], a cytokine driving acute inflammation and apoptosis, also decreases [52]. Iron accumulation, combined with macrophage‐ and neutrophil‐derived H₂O₂, catalyzes the formation of hydroxyl radicals (OH) via the Fenton reaction, causing oxidative damage and perpetuating inflammation [53, 54]. Excess Fe²⁺ in chronic wound exudates also promotes the activation of pro‐inflammatory macrophages and senescent fibroblasts, key drivers of non‐healing states [54, 55]. Some agents like EDTA can also regulate inflammation by chelating iron, especially in chronic venous ulcers (CVUs) [48, 54], where venous stasis and erythrocyte extravasation lead to iron overload in tissue and wound exudates [56].

### 2.5 Addressing MMPs Hyperactivity

MMPs are crucial for ECM remodeling in wound healing [57], but their excessive and uncontrolled activity in chronic wounds impairs healing [58]. Elevated MMP levels degrade essential matrix components like collagen, elastin, and fibronectin, causing wound enlargement [57, 58, 59]. They also hinder cell proliferation by breaking down interleukins, cytokines, and growth factors vital for healing [59]. Since MMPs rely on cations like Zn²⁺ and Mg²⁺ as cofactors [60, 61], chelating agents, such as EDTA, EGTA, DMSA, DMPS, lipoic acid, and oxalate salts can inhibit their activity. Among these, EDTA is particularly effective, acting as a broad‐spectrum MMP inhibitor [61] and offering additional benefits. Acidification of the wound bed can complement EDTA by reducing MMP activity, which peaks at alkaline pH levels (typically > 8) [41, 60].

### 2.6 Addressing Oxidative Stress

Numerous studies confirm the role of oxidative stress in chronic ulcers' pathogenesis, where excessive reactive oxygen species (ROS) from respiratory bursts and phagocyte lysis delay healing through cytotoxic effects [39, 54, 62, 63]. To accelerate healing, reducing oxidative stress via antioxidants or free radical scavengers is essential [62, 63, 64]. Hydrophilic reducing agents, such as ascorbic acid, vitamin E, oxalate salts, uric acid, and lipoic acid are potential candidates, with ascorbic acid being the most suitable due to its strong antioxidant properties and additional benefits for chronic wound treatment [65, 66, 67]. Ascorbic acid can also regulate inflammation via various mechanisms [67, 68, 69, 70, 71], serves as an essential cofactor for key enzymatic reactions in healing [71], and promotes anti‐inflammatory effects in macrophages while suppressing pro‐inflammatory processes [68]. Additionally, it regulates neutrophil apoptosis, protecting tissues from oxidative damage during respiratory bursts [70].

### 2.7 Addressing Hypoxia

Acidification of the wound also enhances oxygenation. A decrease of just 0.6 pH units has been shown to increase oxygen release from hemoglobin to the wound by nearly 50% [72]. Since the likelihood of scarring is low when tissue oxygen tension is < 20 mmHg and high when it exceeds 40 mmHg, acidification can further support healing in this aspect [41, 42]. As we propose using lactic acid for wound acidification, it is important to note that lactic acid itself promotes healing by improving oxygen supply to chronic wounds. First, lactate stimulates angiogenesis by increasing Vascular Endothelial Growth Factor (VEGF) expression in macrophages [73, 74, 75]. Second, lactate is a pH‐independent vasodilator through stimulation of Nitric Oxide Sinthase (eNOS) [73, 76].

### 2.8 Addressing Chronic Pain

Chronic wound pain arises from two main mechanisms: nociceptive pain, triggered by inflammatory mediators stimulating afferent receptors and causing sharp, stabbing, or aching sensations [77, 78]; and neuropathic pain, resulting from nerve damage, often described as burning or stinging, with intensity unrelated to visible tissue damage [79].

Many patients experience mixed pain, where inflammation heightens sensitivity (hyperalgesia), repeated stimuli cause allodynia, and ectopic nerve firing produces shock‐like sensations, all of which can be worsened by infection or maceration [80].

Chronic pain elevates cortisol and vasopressin levels, promoting infection via immunosuppression and hypoxia through vasoconstriction [19]. Thus, effective pain management is essential, improving both quality of life and wound healing rates.

A systematic review identified lidocaine/prilocaine cream, ibuprofen foam, and morphine gel as effective topical agents [81, 82, 83, 84]. To align with the principle of multifunctional components, phenytoin is recommended for its dual benefits: topical analgesia for neuropathic pain and enhanced wound healing [85, 86]. Alternatives include barbiturates [87] and ethosuximide [88], structural analogs of phenytoin. Ethosuximide shows analgesic effects in models of neuropathic and nociceptive pain [88, 89], and promotes healing by enhancing collagenization [90].

### 2.9 Addressing Lack of Cellular Proliferation and Extracellular Matrix Formation

The healing process is divided into four overlapping phases: hemostasis, inflammation, proliferation, and remodeling [90]. Chronic wounds are stalled in the inflammatory phase, so effective treatment must shift the wound from this phase while also promoting the proliferative phase [90]. In this regard, acidification can also promote granulation tissue formation and re‐epithelialization [9, 41]. For example, acidifying the wound bed stimulates fibroblast proliferation and overall cell multiplication, particularly at a pH of 6.8–7.2 [9, 41, 43]. Fibroblasts synthesize collagen and elastin, essentials for ECM formation [91]. Acidification also protects newly formed elastin from degradation by elastase, whose activity peaks at pH 8.3 [40, 42]. Lactic acid, proposed for acidification, further enhances collagen synthesis by activating fibroblast collagen promoters [73]. Additionally, ascorbic acid, previously proposed for its antioxidant properties, is also essential for collagen and elastin synthesis [92]. On the other hand, lactic acid also promotes endothelial proliferation and angiogenesis [73, 74] which is essential for granulation tissue formation and healing [91]. Acidification also enhances keratinocyte proliferation, differentiation, and maturation [43] and inhibits serine proteases, which degrade the corneodesmosomes, the structures that hold keratinocytes together [93, 94, 95].

### 2.10 Addressing Chronic Infection

The microenvironment of chronic wounds, characterized by necrotic debris, hypoxia, and a sustained but dampened immune response, fosters bacterial growth [90, 91, 96]. Bacteria evolve along a continuum from contamination to colonization, critical colonization, and infection [97]. Once present, they adopt two phenotypes: planktonic [96, 97] (free‐living, metabolically active, producing virulence factors, but more antibiotic‐susceptible and capable of triggering inflammation) or biofilm‐associated. Biofilm development begins when planktonic bacteria adhere to wound surfaces through physicochemical and electrostatic interactions [98, 99], followed by genetic reprogramming and ECM production [99]. The ECM, composed mainly of extracellular polymeric substances (EPS—polysaccharides, proteins, lipids, and eDNA), accounts for 90% of biofilm volume and provides adhesion, cohesion, protection, and redox capacity [99, 100, 101, 102, 103]. eDNA, derived from bacterial secretion or host cell lysis, is a ubiquitous structural component [103]. Given the strong association between biofilms and delayed healing [104], management must integrate biofilm inhibition and disruption.

Biofilm disruption is more challenging due to polymicrobial variability in EPS composition [99, 101]. Approaches include targeting EPSs and modifying physicochemical factors, such as electrostatics, wettability, stiffness, pH, surface tension, and cation concentration [105, 106]. EPS destabilization by DNase (e.g., Dornase alfa [107, 108, 109, 110, 111]), cation chelation (e.g., EDTA [48]), surfactants (e.g., Polysorbate 80 [106, 112, 113]), or acidic pH (e.g., lactic acid [41, 45, 114, 115]) can disrupt biofilms [101, 102, 103, 104, 105, 106, 116, 117]. Moreover, a multitargeted approach—combining a DNase, a chelating agent, a surfactant, and acidification—could maximize disruption effectiveness and spectrum.

Biofilm inhibition targets planktonic bacteria and prevents adhesion, since up to 30% of biofilm populations revert daily to this state [21]. Strategies include altering wound bed conditions and applying bacteriostatic/bactericidal agents. Lactic acid, due to its low pKa and hydrophobicity, penetrates membranes and induces acid stress, ATP depletion, metabolic shifts, and free radical damage, ultimately suppressing adaptation [97, 118, 119, 120]. Sensitization by lactic acid increases bacterial susceptibility to detergents (Polysorbate 80) [89] or antimicrobials (EDTA) [48, 120]. Polysorbate 80 further reduces nutrient availability, increases membrane permeability, and transports ions [106, 112]. EDTA destabilizes Gram‐negative membranes by removing divalent cations linking lipopolysaccharides, releasing LPS, and exposing phospholipids, thereby inhibiting growth [40, 48, 121].

### 2.11 Addressing Excessive Exudation

In chronic ulcers, persistent inflammation increases vasodilation and vessel permeability, leading to continuous extracellular fluid buildup and prolonged exudate production [22]. Managing exudate is crucial in treating chronic wounds, as excessive amounts can cause periwound maceration, and its composition is harmful to healing [22, 122]. However, maintaining optimal moisture levels is equally important to support cell migration and promote healing [122]. Therefore, the optimal strategy is to absorb excess exudate while maintaining moisture balance.

Various biomaterials have been proposed for their water‐absorbing capabilities [122, 123]. However, selective water removal can concentrate solutes like proteins and enzymes, increasing oncotic pressure and further promoting exudate production and its harmful effects [124]. Hydrogel‐based wound dressings are highly effective for managing exudate and maintaining moisture in chronic wounds [125]. These hydrophilic, three‐dimensional polymer networks are crosslinked chemically or physically and can absorb significant amounts of water and solutes while retaining their structural integrity [125]. Hydrogels create a moist environment for dry wounds and absorb excess exudate in overly wet wounds [124, 125]. They also offer adhesion‐free coverage, pain relief through cooling, active participation in healing, and ease of use [125]. Polysaccharide‐based hydrogels, including those made from starch, dextran, chitosan, carrageenan, alginate, and cellulose, are particularly suitable for chronic wound care due to their biodegradability, versatility, hydrophilicity, and availability [124, 125]. Among these, cellulose‐based hydrogels stand out for their biocompatibility, non‐toxicity, non‐immunogenicity, high absorption capacity, low cost, and excellent thermal and chemical stability [125].

### 2.12 Addressing Necrotic Tissue Accumulation

The primary goal of debridement is to remove devitalized tissue, including necrotic tissue, slough, bioburden, biofilm, and apoptotic cells, to expose healthy, well‐perfused tissue that supports epithelial cell migration and wound healing [126]. Debridement methods include autolytic, biological, enzymatic, surgical, and mechanical approaches, chosen based on wound characteristics [126]. Key components removed during debridement are retained in a superficial gel layer composed mainly of DNA from necrotic/apoptotic cells and fibrin from the coagulation and inflammatory cascade [122, 126]. We propose an alternative debridement approach that combines DNA degradation through DNase activity with the inhibition of fibrin formation. Calcium, essential for the coagulation cascade as a cofactor for enzymes like thrombin, can be targeted using chelators like EDTA, which act as anticoagulants [48]. Additionally, as thrombin's activity is optimal at pH 8.0–8.5, wound acidification could further regulate fibrin clot formation. Combining DNase and EDTA at acidic pH could potentially enhance debridement and promote healing by reducing fibrin, a physical barrier that hinders the process. Autolytic debridement, reliant on enzymes, such as hyaluronidases, SC thiol proteases, and DNases, is often impaired in chronic wounds due to their alkaline pH. Therefore, wound acidification could also restore normal autolytic debridement and improve healing outcomes [40].

### 2.13 Consequences of the Hypothesis and Discussion

Our evaluation of the hypothesis indicates that it is indeed feasible to address all CIFs while simultaneously meeting therapeutic needs (Table 1) through a rational combination of functional components: an organic carboxylic acid, a chelating agent, a free‐radical scavenger, a structural analog of phenytoin with analgesic/anesthetic properties, a non‐ionic surfactant, and an enzyme (DNase I) with optimal activity at acidic pH. While several molecules could theoretically fulfill these roles, our selection was guided by a systematic evaluation of both the hypothesis principles and key pharmaceutical development criteria. Specifically, we prioritized: (1) multifunctionality and the potential to generate synergistic effects when combined; (2) the availability of robust regulatory information, including well‐characterized pharmacokinetics, pharmacodynamics, and safety profiles; (3) favorable physicochemical properties, such as solubility, stability, and activity under acidic pH; and (4) pharmaco‐technical compatibility with semisolid formulations. When assessed against these parameters, lactic acid, EDTA, ascorbic acid, ethosuximide, polysorbate 80, and hr‐Dornase alfa consistently emerged as the most suitable representatives, balancing therapeutic relevance with translational feasibility.

These molecules also stand out because they are FDA‐approved compounds with established safety and low systemic and topical toxicity [95]. Such regulatory status significantly lowers the translational barrier, strengthening the plausibility of the proposed approach. On this basis, we argue that a topical combination product, formulated as a cellulose‐based hydrogel incorporating these agents, represents a compelling and innovative strategy for chronic wound management. While experimental validation is still required, the convergence of mechanistic rationale, regulatory feasibility, and safety profiles strongly suggests that this hypothesis could pave the way for a clinically viable, comprehensive therapeutic solution.

Having identified the most promising molecules, the next step would be to explore whether each agent retains its previously demonstrated therapeutic activity once combined. The first logical preclinical studies could include: (1) testing whether EDTA maintains its MMP–inhibitory activity at formulation‐relevant concentrations (in vitro MMP assays); (2) assessing whether ascorbic acid preserves its antioxidant capacity (e.g., DPPH, ORAC, or comparable assays); (3) evaluating whether lactic acid stimulates VEGF expression in vitro (qPCR/ELISA) and promotes angiogenesis in vivo (e.g., rodent wound or CAM assays); (4) investigating whether the molecular combination exerts synergistic inhibition and disruption of biofilms formed by bacterial species commonly isolated from chronic wounds (using standard in vitro biofilm models, such as MBEC, crystal violet, CFU counts, and viability assays); and (5) validating these findings in animal models of infected chronic wounds, assessing impacts on biofilm burden, wound healing dynamics, and safety. Further exploratory studies could enhance translational feasibility, including: (1) physicochemical stability and compatibility testing under formulation‐relevant conditions (pH, temperature, and ionic strength) to ensure therapeutic activities are not compromised; (2) preliminary safety assessments in skin‐relevant cell lines (keratinocytes, fibroblasts, and macrophages) to verify the absence of cytotoxicity within therapeutic ranges; (3) quantitative synergy analyses (checkerboard or Chou–Talalay methods) to determine whether the combination yields additive or synergistic effects; and (4) preformulation release studies to evaluate how these molecules behave when incorporated into a cellulose‐based hydrogel, linking preclinical activity with the intended topical application.

All these experiments could be conducted within concentration ranges already shown to produce therapeutic effects individually, providing a rational framework to test activity retention, compatibility, and potential synergism in combination.

## 3 Limitations

While the proposed combination of FDA‐approved molecules is mechanistically and conceptually promising, this study is purely hypothetical and lacks experimental validation. Key limitations include the unknown retention of individual activities within the combination, potential interactions that may be synergistic or antagonistic, and untested stability and compatibility in a cellulose‐based hydrogel formulation. Additionally, although each molecule has an established safety profile individually, the safety and cytotoxicity of the combined formulation have not yet been assessed in relevant cellular or animal models. These limitations highlight the need for comprehensive preclinical studies before clinical translation can be considered.

## 4 Conclusion

This hypothesis proposes that a rationally designed combination of well‐characterized, FDA‐approved molecules—each targeting distinct CIFs—could provide an integrated therapeutic strategy for chronic wounds. By leveraging multifunctionality, potential synergistic interactions, and pharmaco‐technical feasibility, such an approach may overcome current limitations in wound management. While the concept remains to be validated experimentally, the convergence of mechanistic rationale, regulatory familiarity, and translational plausibility makes this a compelling avenue for future investigation, with the potential to significantly improve clinical outcomes in patients with chronic wounds.

## Author Contributions

**Alberto Nicolás Ramos:** writing – original draft, conceptualization, visualization, writing – review and editing, investigation, funding acquisition, validation. **Nicolás Cerusico:** conceptualization, investigation, funding acquisition, writing – original draft, validation, visualization, writing – review and editing. **Romina Chavez‐Jara:** conceptualization, investigation, funding acquisition, writing – original draft, validation, visualization, writing – review and editing. All authors have read and approved the final version of the manuscript. Romina Chavez‐Jara, the corresponding author, had full access to all the data in this study and takes complete responsibility for the integrity of the data and the accuracy of the data analysis.

## Disclosure

The lead author Romina Chavez‐Jara affirms that this manuscript is an honest, accurate, and transparent account of the study being reported; that no important aspects of the study have been omitted; and that any discrepancies from the study as planned (and, if relevant, registered) have been explained.

## Conflicts of Interest

The authors declare no conflicts of interest.

## Data Availability

Data sharing is not applicable to this article, as no new data were created or analyzed in this study.
